# Structural and mechanical properties of Si-doped CrN coatings deposited by magnetron sputtering technique

**DOI:** 10.1016/j.heliyon.2023.e13461

**Published:** 2023-02-03

**Authors:** Anand Vyas, Ahmed Aliyu

**Affiliations:** a Division of Science, Engineering and Health Studies, SPEED, The Hong Kong Polytechnic University, Hong Kong, China; b Department of Chemical Engineering, Federal University Wukari, Taraba State, Nigeria; c Department of Materials Engineering, Indian Institute of Science, Bangalore, India

**Keywords:** Magnetron sputtering, CrN coatings, CrSiN coating, Si (100) substrate, Microstructure, Mechanical properties, CrN, Chromium nitride, CrSiN, Chromium silicon nitride, Si, Silicon, SEM, Scanning electron microscopy, XRD, X-ray diffraction, AFM, Atomic force microscopy, XPS, X-ray photoelectron spectroscopy

## Abstract

This study successfully deposited Si-doped CrN coatings onto Si (100) substrate by direct current magnetron sputtering. The concentration of Si in the CrSiN coatings was varied by changing the Si target current during deposition. The microstructural and mechanical properties were determined by employing X-ray diffraction, X-ray photoelectron spectroscopy, atomic force microscopy, and nanoindentation test. According to the results, the coating with 3.3 at.% Si contents (CrSiN-2) show an increase and decrease in the crystallite size and coating surface roughness, respectively, leading to solid solution hardening with an optimum hardness and elastic modulus of 21.37 GPa and 205.68 GPa, respectively. With continued Si addition, the coating roughness increased and the mechanical properties gradually decreased and attained 184.08 GPa and 18.95 GPa for the elastic modulus and hardness of the coating with a maximum Si concentration of 9.2 at.% (CrSiN-5).

## Highlights

• CrSiN coatings was deposited on Si (100) substrate by direct current magnetron sputtering.
• Si content within the coatings was varied by changing the Si target current from 2.0 to 5.0 A.
• CrSiN-2 coating with 3.3 at.% Si content exhibited the optimum hardness and elastic modulus.

## Graphical abstract

**Figure:**
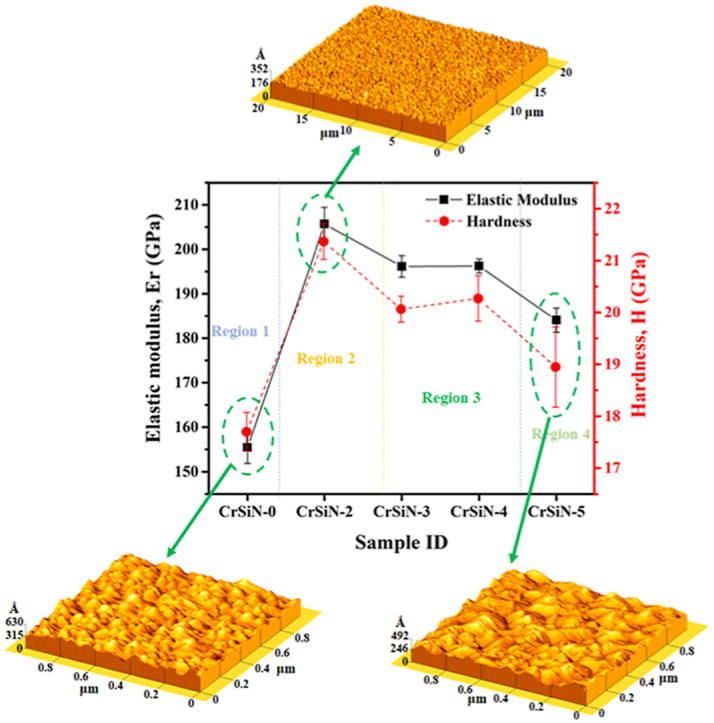

## 1 Introduction

Over the years, chromium nitride (CrN) coatings have been reportedly used in casting and tribological forming applications due to the coating's superior wear resistance as a result of its low friction coefficient and relatively high hardness [[1], [2], [3]]. However, the CrN coatings are difficult to apply to high-speed applications because of their low hardness compared to the TiN coatings. Therefore, incorporating an element such as Al, Si, C, etc., into the CrN matrix to form a ternary CrXN (where X = Al, Si, C, etc.) coating systems have been actively explored to enhance the properties of the coatings materials further [4,5].

Among these elements, incorporating Si atoms into CrN coatings in forming ternary chromium silicon nitride (CrSiN) coating led to a significant increase in their mechanical properties, due to forming a fine microstructure and formation of SiN amorphous phase at the grain boundary [2,5,6]. The strengthening mechanisms for the incorporation of Si into CrN coatings include solid-solution strengthening, structural hardening due to the formation of a nanocomposite, and the influence of the residual stress [2,6]. For instance, Chang et al. [5] reported a low nanoindentation hardness of 5.9 GPa for a Cr_50_N_50_ film with a face-centered cubic (fcc) structure, whereas the hardness increased to 17 GPa for a CrSiN film with 14 at.% Si content because of the formation of a nanocomposite structure. In the same studies, they reported that beyond 16 at.% Si content, the hardness decreases to 14 GPa because of the formation of a high volume fraction of an amorphous SN_x_ structure [5]. Lee et al. [7] also study the formation and properties of CrSiN thin film using closed-field unbalanced magnetron sputtering. Their study reported that the microstructure and hardness of CrSiN film depend on the Si concentration, which is anticipated from the solid solution hardening as a result of the substitution of the Cr atom site for the Si atom site. In addition, much research has shown that the Si concentration in CrSiN coatings can significantly impact their microstructure and properties [8,9]. Furthermore, it is important to note that the solubility of Si in transition metal nitride coatings and the affinity of N to the transition metal element differ, which can prevent the formation of a typical nanocomposite structure.

However, the literature on the effect of Si doping on CrN coating using direct current magnetron sputtering by varying the Si target current while keeping the Cr target current and N_2_ flowrate constant is scant due to the fact that the results of the previous investigation do not coincide with each other. Therefore, investigating the effects of Si doping to better understand the microstructure, phase component, and correlations between Si concentration and mechanical properties of CrN coatings is crucial. Thus, in this study, CrSiN with different Si elemental compositions were deposited on Si (100) substrate through direct current magnetron sputtering. The coating microstructure was studied using atomic force microscopy, X-ray diffraction, and X-ray photoelectron spectroscopy techniques. While the mechanical properties were determined using the nanoindentation technique.

## 2 Experiment

### 2.1 Deposition of CrSiN coatings using direct current magnetron sputtering

The nanocomposite film deposition was performed in a reactive close-field unbalanced dc-magnetron sputtering system (UDP450, Teer Coating Limited) on Si (100) substrate. Two Cr and one Si, target (99% pure) were used as cathode materials for depositing nanocomposite Cr–Si–N_*x*_ thin films. The sputtering was carried out at a bias voltage of −80 V with a substrate rotation speed of 10 rpm. Before sputtering the background pressure was pumped down to 2.7 × 10^−4^ Pa and the working pressure, consisting of a constant flow rate of Ar and reactive gas N_2_ was set at 0.26 Pa during all depositions. To control the partial pressure of reactive gas N_2_ a built-in closed-loop optical emission monitor was employed. The Cr current was fixed at 5.0 A, whereas the Si target current was varied at 0.0, 2.0, 3.0, 4.0 and 5.0 A respectively. The substrate surface was pre-sputtered with Ar plasma at a negative bias voltage of 500 V for 30 min before the film deposition, followed by the deposition of a thin Cr buffer layer (∼100 nm in thickness) to reduce stress at the interface with the substrate. During deposition, the substrate temperature was ∼200 °C.

### 2.2 Characterization

**Fig. 1:**
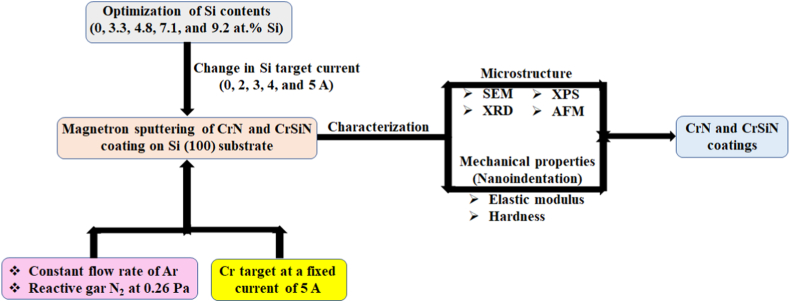
Schematic diagram of the CrN and CrSiN coatings.

The elemental concentration of the films was determined by X-ray photoelectron microscopy (XPS, PHI 5802 system) with a monochromatic Al Kα X-ray source (hν = 1486.6 eV). The crystallographic structure of the films was determined by XRD using a Rigaku MiniFlex diffractometer with a Cu tube operated at 40 kV and 30 mA. The measurements were carried out using Cu Kα radiation with a Ni filter to remove Cu Kβ reflections. The surface topography of the films was acquired by scanning the samples in the air with AFM (Auto-Probe CP, Park Scientific Instruments) operated in a contact mode. The scan areas are 1 × 1 μm^2^ with a resolution of 256 × 256 pixels. The nano-hardness and elastic modulus were analyzed by nanoindentation measurements with a maximum load of 20 mN. Ten separate measurements were taken for each sample to get a mean value. In order to minimize the influence of roughness and substrate the maximum indentation depth was kept to less than 10% of the total film thickness. The schematic diagram of the coatings process in this study is shown in Fig. 1.

## 3 Results and discussion

**Fig. 2:**
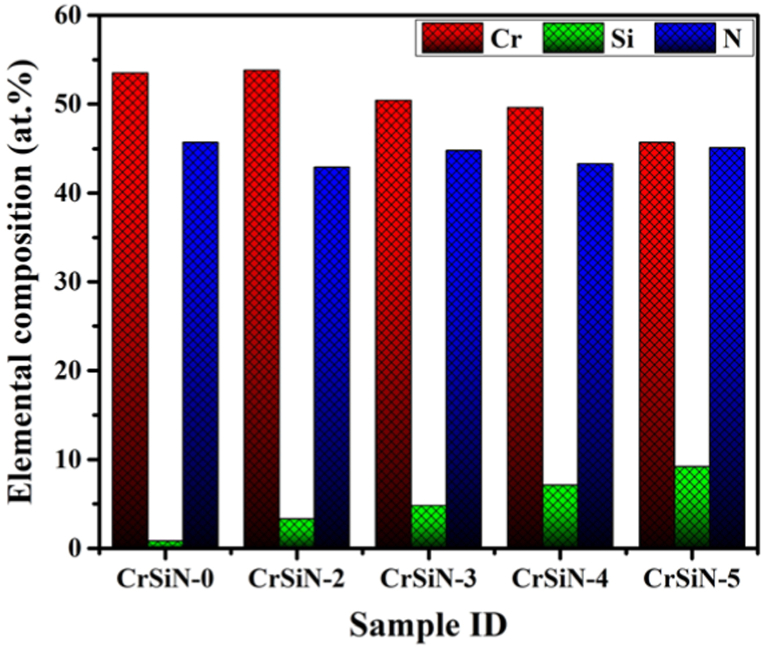
Elemental composition of CrSiN coatings as a function of the Si contents.

Fig. 2 shows the elemental composition of the CrSiN coatings at different Si contents by changing the Si target current from 2.0 to 5.0 A. The Cr and Si content within the coatings decreased and increased, respectively, with an increase in Si target deposition current. The Cr content decreased from 53.5 to 45.7 at.%, while the Si content increased from 0.8 to 9.2 at.%. The N content in the coatings were 45.7, 42.9, 44.8, 43.3, and 45.1 at.% for CrSiN-0, CrSiN-2, CrSiN-3, CrSiN-4, and CrSiN-5 coating, respectively, which implies that the N content within the coatings did not follow any particular trend. Also, the observed different variation tendencies of the N contents in the coatings suggest differences in affinity between the N and Cr or Si elements. The observed 0.8 at.% Si in the pristine coating (i.e., CrSiN-0) probably comes from the Si (100) substrate.

**Fig. 3:**
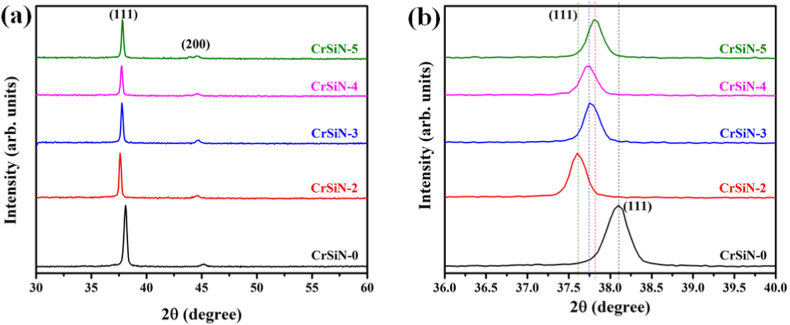
(a) XRD patterns of CrSiN coatings as a function of the Si contents, and (b) XRD patterns for 2 angle range of 36o-40o showing a shift in (111) diffraction angle of the coatings.

**Fig. 4:**
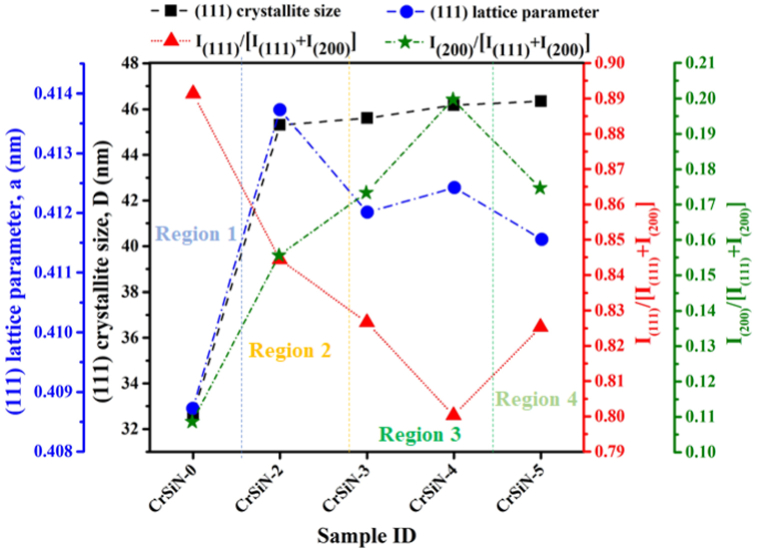
Correlation between the crystallite size, lattice parameters, and coatings growth of CrSiN coatings as a function of the Si contents.

Fig. 3 shows the X-ray diffraction (XRD) patterns of CrSiN coatings at different Si contents by changing the Si target current from 2.0 to 5.0 A. The XRD patterns of the CrSiN-0 coating in Fig. 3(a) showed diffraction peaks at 38.10° and 45.20°, corresponding to the orientations of (111) and (200) crystal planes (JCPDS #08-7235). Compared to the pristine CrSiN-0 coating, the diffraction peaks of the CrSiN-2 coating (i.e., 3.3 at.% Si) shift to a lower angular position which could be attributed to the intrinsic compressive stress of the coating or the CrN lattice expansion [8,10]. On the contrary, the diffraction peaks tend to shift to higher diffraction angles for the CrSiN-3 (i.e., 4.8 at.%), CrSiN-4 (i.e., 7.1 at.%), and CrSiN-5 (i.e., 9.2 at.%) coatings with respect to the CrSiN-2 coating, suggesting intrinsic tensile stress in the coating and a decrease in lattice parameter (see, Fig. 3(b)) due to substitution of some Cr-atoms (with atomic radius: 128 p.m.) by Si-atom (with atomic radius: 117.6 p.m.) in the lattice [[11], [12], [13]]. The calculated lattice parameters in Fig. 4 show that the Si addition initially increases the lattice parameter and later decreases at high Si contents (but higher than that of the pristine CrSiN-0 coating), suggesting a lattice distortion of the coating crystal phase. The variation of the corresponding I_(111)_/[I_(111)_ + I_(200)_] and I_(200)_/[I_(111)_ + I_(200)_] ratios as a function of Si contents in CrSiN-0 coating are shown in Fig. 4. The results show that the increase in Si content favors the coating growth of the (200) plane and decreases the (111) peak intensity, implying that the Si addition causes strong changes in the coating's microstructural evolution. Furthermore, the values of the full width at half maximum (fwhm) of the most intense XRD peak (i.e., (111) peak) were used to calculate the crystallite size (D_(111)_) using Scherrer's equation [14], and the results were shown in Fig. 4. The results show that the (D_(111)_) increases for the CrSiN-2 (3.3 at.% Si) coating, then after, the (D_(111)_) is not influenced by the increase in the Si content within the CrN matrix. This observation can be attributed to the solubility of the Si in CrN to form a solid solution.

**Fig. 5:**
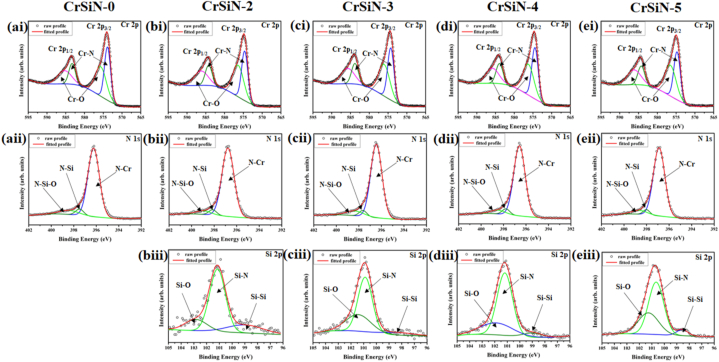
High-resolution XPS spectra of (a1)-(e1) Cr 2p, (aii)-(eii) N 1s, and (biii)-(eiii) Si 2p core level of CrSiN coatings as a function of the Si contents.

Fig. 5 shows the XPS profile of the CrSiN-0, CrSiN-2, CrSiN-3, CrSiN-4, and CrSiN-5 coatings. The Cr 2p, Si 2p, and N 1s signals were recorded for each coatings sample. The Cr signal in Fig. 5(ai), (bi), (ci), d(i), and (ei) shows a spectrum containing two doublets, representing Cr–N and Cr–O bonds [7,15]. The binding energy values of the Cr 2p_2/3_ for the Cr–N in the deposited CrSiN coatings at different Si contents were 574.3–574.7 eV, which is comparable with the report for the Cr–N bonds by Chang et al. [16] in CrSiN films (574.57–574.6 eV). The N 1s signals in Fig. 5(aii), (bii), (cii), (dii), and (eii) were deconvoluted into three parts corresponding to the N–Cr (396.5–396.8 eV), N–Si (397.7–398.2 eV), and N–Si–O (398.4–399.0 eV) bonds. Studies [[17], [18], [19], [20], [21]] have shown binding energies of 396.4–396.8, 397.0–397.5, and 399.0–399.9 eV for the N–Cr, N–Si, and N–Si–O bonds, respectively. The Si signals in Fig. 5(biii), (ciii), d(iii), and (eiii) were deconvoluted into three, namely, Si–Si, Si–N, and Si–O bonds with corresponding bindings energy values of 98.2–98.9, 101.1–101.6, and 102–102.9 eV, respectively, which are comparable with the corresponding values of 99.0–99.5 [16,18], 101.5–101.8 eV [16], and 103.0–103.5 eV [18,19], respectively, reported in the literature. Also, it was observed that the intensity ratio of the N–Cr: N–Si varies as the Si content within the coatings increases from 0.8 at.% (pristine CrSiN-0) to 9.2 at.% (CrSiN-5).

**Fig. 6:**
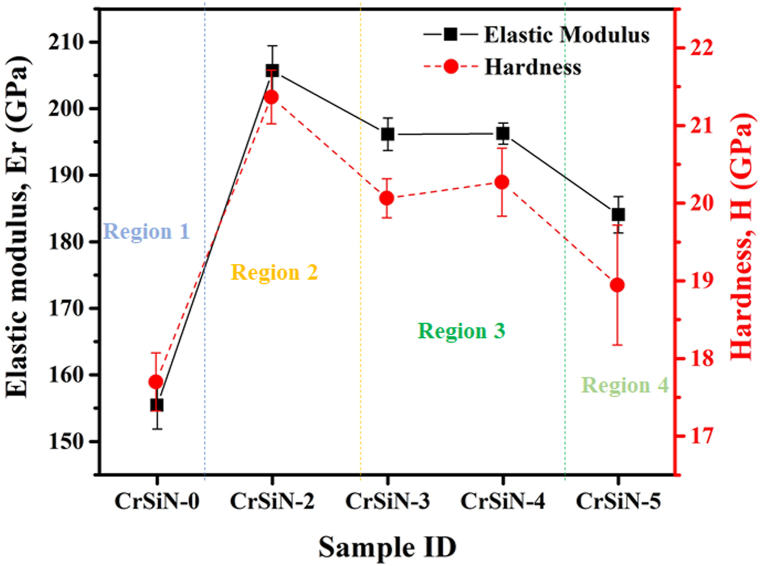
Elastic modulus and hardness of CrSiN coatings as a function of the Si contents.

Fig. 6 shows the elastic modulus and hardness of the CrSiN coatings as a function of the Si contents by changing the Si target current. As the Si contents within the coating matrix increase, the coating hardness and elastic modulus increase from ∼17.70 GPa to ∼155.42 GPa for CrSiN-0 coating to a maximum value of ∼21.37 GPa and 205.68 GPa for CrSiN-2 (3.3 at.% Si) coating. These changes in the coating hardness and elastic modulus of the pristine CrSiN-0 coating as a result of the 3.3 at.% Si addition can be attributed to the microstructural changes of the CrN coatings in its solid solution hardening of the crystallite and an increase in residual stress of the coating as a result of lattice distortion by the Si addition as shown in Fig. 3(b). With further increase in the Si contents (i.e., for CrSiN-3 (4.8 at.% Si), CrSiN-4 (7.1 at.% Si), and CrSiN-5 (9.2 at.% Si) coatings) the hardness of the coatings and elastic modulus steeply decreases and this can be attributed to the possible high volume fraction of the amorphous phase within the CrSiN-0 coating matrix. It has been reported that an increase in the volume fraction of an amorphous phase within the CrSiN matrix phase resulted in hardness and elastic modulus reduction [22].

**Fig. 7:**
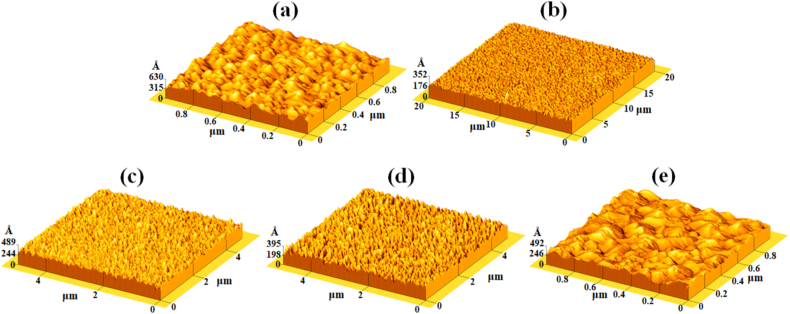
AFM images of (a) CrSiN-0, (b) CrSiN-2, (c) CrSiN-3, (d) CrSiN-4, and (e) CrSiN-5 coatings on Si (100) substrate with different Si contents.

**Fig. 8:**
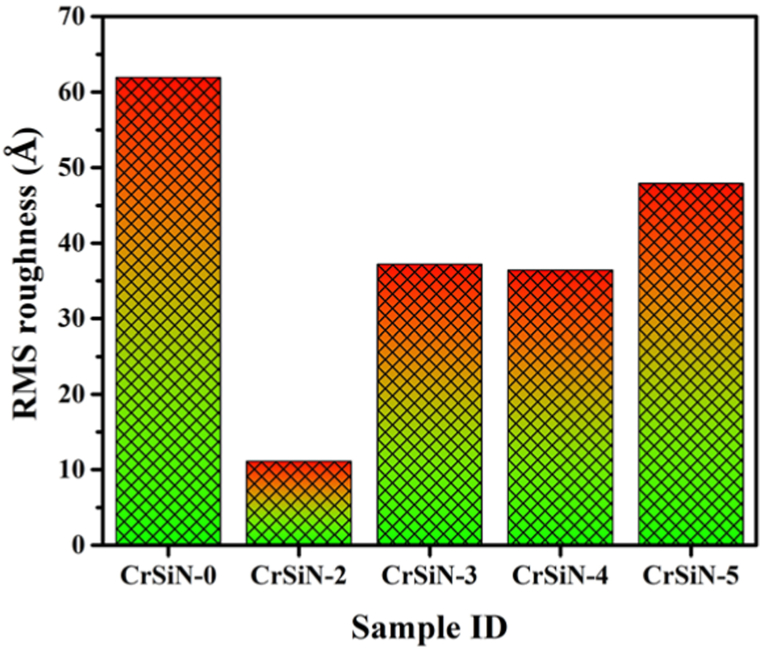
RMS roughness of CrSiN coatings as a function of the Si contents extracted from the AFM images in Fig. 6.

**Fig. 9:**
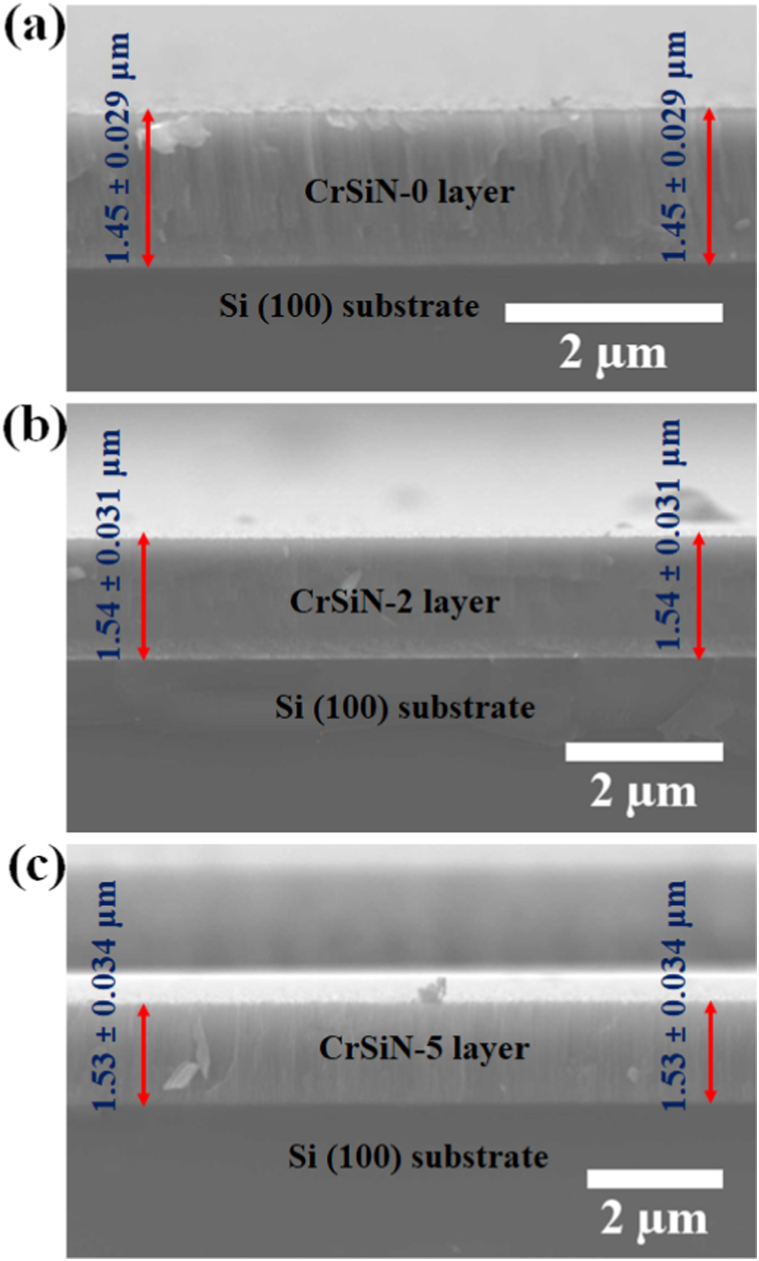
SEM cross-section micrographs of (a) CrSiN-0, (b) CrSiN-2, and (c) CrSiN-5 coatings.

The AFM surface morphologies images of the pristine CrSiN-0 and CrSiN coatings with different Si contents by varying the Si target current from 2.0 to 5.0 A are shown in Fig. 7(a)–(e). While the RMS roughness of the coatings extracted from the AFM images in Fig. 7(a)–(e) are presented in Fig. 8. It is observed from the figures (i.e., Fig. 8) that the surface roughness of the coatings decreases from 61.9 to 11.1 Å for CrSiN-0 and CrSiN-2 (3.3 at.% Si) coating (i.e., Fig. 7(a) and (b)), respectively. This implies that Si incorporation into the CrN matrix tends to make the coating surface morphology smother by changing the coating microstructure to a finer composite that is capable of enhancing the coating hardness and elastic modulus. However, the coatings surface roughness increased to 37.2, 36.4, and 47.9 Å, for CrSiN-3 (4.8 at.% Si), CrSiN-4 (7.1 at.% Si), and CrSiN-5 (9.2 at.% Si) coatings (i.e., Fig. 7(c), (d), and (e)), respective. Therefore, the coating surface roughness was considerably affected by the Si content in CrN coating. These results agree with the work of Kim et al. [23], they reported an optimum hardness for a fine composite CrSiN coating due to the formation of an amorphous Si_3_N_4_ capable of causing grain boundary hardening and, consequently, enhancing cohesive energy of the interphase boundaries within the coatings [23]. Furthermore, Fig. 9(a)-(c) shows the cross-sectional micrograph obtained from the SEM of the CrSiN-0, CrSiN-2, and CrSiN-5 coatings, respectively, where the Si (100) substrate region and the coating region have been identified. From the cross-sectional images, the average coatings thickness of the fabricated film of the CrSiN-0, CrSiN-2, and CrSiN-5 coatings were obtained to be 1.45 ± 0.029 μm (i.e., Fig. 9(a)), 1.54 ± 0.031 μm (i.e., Fig. 9(b)), and 1.53 ± 0.034 μm (i.e., Fig. 9(c)), respectively.

## 4 Conclusion

The CrSiN coatings have been successfully deposited on a Si (100) substrate by changing the Si target current at 0.0, 2.0. 3.0. 4.0 and 5.0 A, respectively, while keeping other parameters constant using the direct current magnetron sputtering technique and systematically studying their microstructure and mechanical properties. Incorporating Si element into the CrN lattice causes the coating diffraction peak to shift to the left and later to the right, suggesting that Si atoms at 3.3 at.% cause intrinsic compressive stress and above 3.3 at.% Si resulted in intrinsic tensile stress on the CrSiN-3 (i.e., 4.8 at.% Si), CrSiN-4 (i.e., 7.1 at.% Si), and CrSiN-5 (i.e., 9.2 at.% Si) coatings. For a 3.3 at.% Si contents, the coating crystallite size and surface roughness increase, and decrease, respectively, leading to solid solution hardening with optimum hardness and elastic modulus 21.37 GPa and 205.68 GPa, respectively. Therefore, this study suggests that the microstructure and mechanical properties of CrSiN-0 coating using direct current magnetron sputtering can thus be significantly enhanced by adding an optimum Si element into the CrN matrix, rendering the coating very attractive for casting and tribological applications.

## Author contribution statement

Anand Vyas: Conceived and designed the experiments; Performed the experiments; Contributed reagents, materials, analysis tools or data; Wrote the paper.

Ahmed Aliyu: Analyzed and interpreted the data; Wrote the paper.

## Funding statement

This research did not receive any specific grant from funding agencies in the public, commercial, or not-for-profit sectors.

## Data availability statement

Data will be made available on request.

## Declaration of interest's statement

The authors declare that they have no known competing financial interests or personal relationships that could have appeared to influence the work reported in this paper.

## Abbreviations

CrN: Chromium nitride
CrSiN: Chromium silicon nitride
Si: Silicon
Cr: chromium
N: nitrogen
Ar: argon
O: oxygen
SEM: Scanning electron microscopy
XRD: X-ray diffraction
AFM: Atomic force microscopy
XPS: X-ray photoelectron spectroscopy
A: ampere; at.%, atomic percentage
GPa: Gigapascal
D: crystallite size
