# Supplementary material for: Structural and mechanical properties of Si-doped CrN coatings deposited by magnetron sputtering technique
Source: Heliyon. 2023 Feb 3;9(2):e13461. doi: 10.1016/j.heliyon.2023.e13461 (PMC9929308; doi:10.1016/j.heliyon.2023.e13461)
Supplement: Multimedia component 1 [file mmc1.pdf]

Prime Novelty Statement

Si-doped CrN coatings were deposited onto Si (100) substrate by employing magnetron sputtering technique. CrSiN-2 coating with 3.3 at.% of Si exhibited the optimum hardness and elastic modulus. The optimum mechanical properties is mainly attributed to the coating crystallite size and surface roughness. Moreover, this could give insights on the selection of CrSiN coatings for casting and tribological applications.
